# Genomic characterization of SARS-CoV-2 from Uganda using MinION nanopore sequencing

**DOI:** 10.1038/s41598-023-47379-z

**Published:** 2023-11-22

**Authors:** Praiscillia Kia, Eric Katagirya, Fredrick Elishama Kakembo, Doreen Ato Adera, Moses Luutu Nsubuga, Fahim Yiga, Sharley Melissa Aloyo, Brendah Ronah Aujat, Denis Foe Anguyo, Fred Ashaba Katabazi, Edgar Kigozi, Moses L. Joloba, David Patrick Kateete

**Affiliations:** 1 Department of Immunology and Molecular Biology, School of Biomedical Sciences, College of Health Sciences, Makerere University, Kampala, Uganda; 2 The African Centers of Excellence in Bioinformatics and Date Intensive Sciences, Infectious Disease Institute, College of Health Sciences, Makerere University, Kampala, Uganda; 3 Multifunctional Research Laboratories, Gulu University, Gulu, Uganda; 4 Department of Biology, Muni University, Arua, Uganda

**Keywords:** Microbiology, Virology, SARS-CoV-2, Infectious diseases, Viral infection

## Abstract

SARS-CoV-2 undergoes frequent mutations, affecting COVID-19 diagnostics, transmission and vaccine efficacy. Here, we describe the genetic diversity of 49 SARS-CoV-2 samples from Uganda, collected during the COVID-19 waves of 2020/2021. Overall, the samples were similar to previously reported SARS-CoV-2 from Uganda and the Democratic Republic of Congo (DRC). The main lineages were AY.46 and A.23, which are considered to be Delta SARS-CoV-2 variants. Further, a total of 268 unique single nucleotide variants and 1456 mutations were found, with more than seventy percent mutations in the *ORF1ab* and *S* genes. The most common mutations were 2042C>G (83.4%), 14143C>T (79.5%), 245T>C (65%), and 1129G>T (51%), which occurred in the *S, ORF1ab*, *ORF7a* and *N* genes, respectively. As well, 28 structural variants—21 insertions and 7 deletions, occurred in 16 samples. Our findings point to the possibility that most SARS-CoV-2 infections in Uganda at the time arose from local spread and were not newly imported. Moreover, the relatedness of variants from Uganda and the DRC reflects high human mobility and interaction between the two countries, which is peculiar to this region of the world.

## Introduction

Severe acute respiratory syndrome coronavirus 2 (SARS-CoV-2) is the causative agent of COVID-19, a severe infectious respiratory disease. SARS-CoV-2 is an enveloped, single-stranded, positive-sense RNA virus^1^. It is among the seven human coronaviruses belonging to the genus *Betacoronavirus* and subgenus *Sarbecovirus.* An individual acquires SARS-CoV-2 infection mainly via inhalation of aerosolized droplets, although infection through aerial droplets and contact has been reported^2^.

The genome sequence of SARS-CoV-2 varies between 29.8 kb and 29.9 kb, and has genomic structures similar to those of other coronaviruses^3^. At the 5′-untranslated region (UTR) is the *ORF1ab* gene encoding the ORF1ab polyproteins, which cover more than two thirds of the genome. At the 3′-UTR are genetic elements encoding the spike (S), envelope (E), membrane (M) and nucleocapsid (N) structural proteins^4^.

Globally, as of 23 October 2022, SARS-CoV-2 had infected approximately 624 million people and caused approximately 6.5 million deaths since COVID-19 outbreak in 2019^5^. This situation is dire with the growing evidence on the increase in mutations in SARS-CoV-2^6^, which has resulted in emergence of new variants. The first SARS-CoV-2 variant was detected and reported in the U.K.^7^. Following this, different countries from various regions of the world, including South Africa, Brazil, the USA and India, have identified and reported other unique variants^8^.

To understand the emergence of SARS-CoV-2 variants, several studies have sequenced and analysed SARS-CoV-2 genome sequences using different sequencing technologies and approaches, including Sanger sequencing, next-generation sequencing like illumina-Miseq and Ion Torrent, and Oxford Nanopore Technology such as MinION^4, 9, 10^. This has consequently generated millions of SARS-CoV-2 genomic sequences accessible from public databases such as GISAID. Among those are SARS-CoV-2 sequences from Eastern Africa, which comprises countries like Kenya, Uganda, Tanzania, Rwanda and Burundi^11–13^, among others.

While short-read sequencing technologies like illumina-MiSeq and others allow accurate detection of minor mutations, they are unable to provide complete viral genome analysis in a single read^14^, and they are expensive as they require special infrastructure^15^. On the other hand, Sanger sequencing, a conventional method, is unable to detect minor variants. However, Oxford Nanopore Technology, e.g. the MinION, generates long reads that allow for detection of both single nucleotide and structural variations within a shorter time and it is cost effective^15^.

In this study, we aimed to determine the genomic variation of SARS-CoV-2 circulating in Uganda between April 2020 and July 2021, using MinION Nanopore sequencing. Specifically, we identified single nucleotide and structural variations in SARS-CoV-2 in clinical samples collected between April 2020 and July 2021. We also report on the genetic relatedness between SARS-CoV-2 detected in Uganda, Kenya, Rwanda, Burundi, the Democratic Republic of Congo (DRC) and South Sudan within the same period.

## Results

Fifty-five samples from the same number of COVID-19 confirmed cases from across the country were investigated; all libraries passed the final quality control for sequencing on the MinION. However, of the 55 samples, 49 generated quality sequences (i.e., Phred score of ≥ 20), and GC content that ranged from 38 to 41%. Upon alignment of sequences to the SARS-CoV-2 reference genome sequence (i.e., Wuhan HU-1, 29,903 base pairs), we generated, on average, a total of 246,207 reads per sample with mean alignment of 72.76%.

### Single nucleotide polymorphisms (SNPs)

A total of 268 unique variants and 1456 mutations were identified in the 49 genomes following variant calling using Medaka. Majority of these variants were detected in the *ORF1ab*, *S* and *N* genes, with more mutations detected in the coding regions than in noncoding regions, see Tables 1 and 2. The mutations detected included missense, synonymous, small indels, and intergenic. Stop gain and loss were also detected in the *ORF1ab* gene, Table S1 (Supplementary Information).

**Table 1:** SARS-CoV-2 mutations in coding and noncoding regions of the sequenced genomes.

Region	No. of SNPs/variants (%)	Genome region	Number of mutations (%)
*ORF1ab*	144 (53.7)	415–21,270	705 (48.4)
*S*	42 (15.7)	21,618–25,357	333 (22.9)
*N*	24 (9)	28,311–29,402	100(6.87)
*M*	13 (4.9)	26,692–27,170	75 (5.2)
*ORF7a*	3 (1.2)	27,520–27,752	74 (5.1)
*ORF3a*	6 (2.2)	25,469–26,162	28 (1.9)
*ORF8-N*	2 (0.7)	28,270–28,271	28 (1.9)
*ORF8*	4 (1.5)	28,076–28,247	19 (1.3)
*ORF6*	3 (1.2)	27,259–27,297	9 (0.6)
*N-ORF10*	1 (0.4)	29,543	2 (0.1)
*ORF7b*	1 (0.4)	27,874	1 (0.07)
*ORF1ab-START*	3 (1.2)	71–241	18 (1.2)
*ORF10-END*	22 (8.2)	29,727–29,862	65 (4.46)
Total	268		1456

**Table 2:** The commonest mutations and amino acid changes.

Gene	Variation	Amino acid change	Frequency (%)
*S*	c.2043C>G	p.Pro681Arg	41 (83.4%)
	c.1841A>G	p.Asp614Gly	33 (67.3%)
	c.433C>A	p.Thr478Lys	26 (53.1%)
	c.467_472delAGTTCA		25 (51%)
	c.425G>A	p.Gly142Asp	25 (51%)
	c.56C>G	p.Thr19Arg	24 (50%)
	c.2848G>A	p.Asp950Asn	23 (46.9%)
*ORF7a*	c.245T>C	p.Val82Ala	32 (65.3%)
	c. 359C>T	p.Thr120Ile	23 (46.9%)
*ORF1ab*	c.14143C>T	p.Leu4715Leu	36 (79.6%)
	c.2772C>T	p.Phe924Phe	34 (69.4%)
	c.9764C>T	p.Thr3255Ile	34 (69.4%)
	c.18955C>T	p.Leu6319Leu	30 (61.2%)
	c.20221A>G	p.Ser6741Gly	29 (59.2%)
	c.16994G>T	p.Ser5665Ile	29 (59.2%)
	c.8721C>T	p.Asp2907Asp	27 (55.1%)
	c.3916G>T	p.Ala1306Ser	27 (55.1%)
	c.6859C>T	p.Pro2287Ser	24 (50%)
	c.8788G>T	p.Val2930Leu	23 (46.9%)
*N*	c.1129G>T	p.Asp377Tyr	25 (51%)
	c.90_98delAGAACGCAG	p.Glu31_Ser33del	12 (24.5%)

### Structural variations

**Figure 1:**
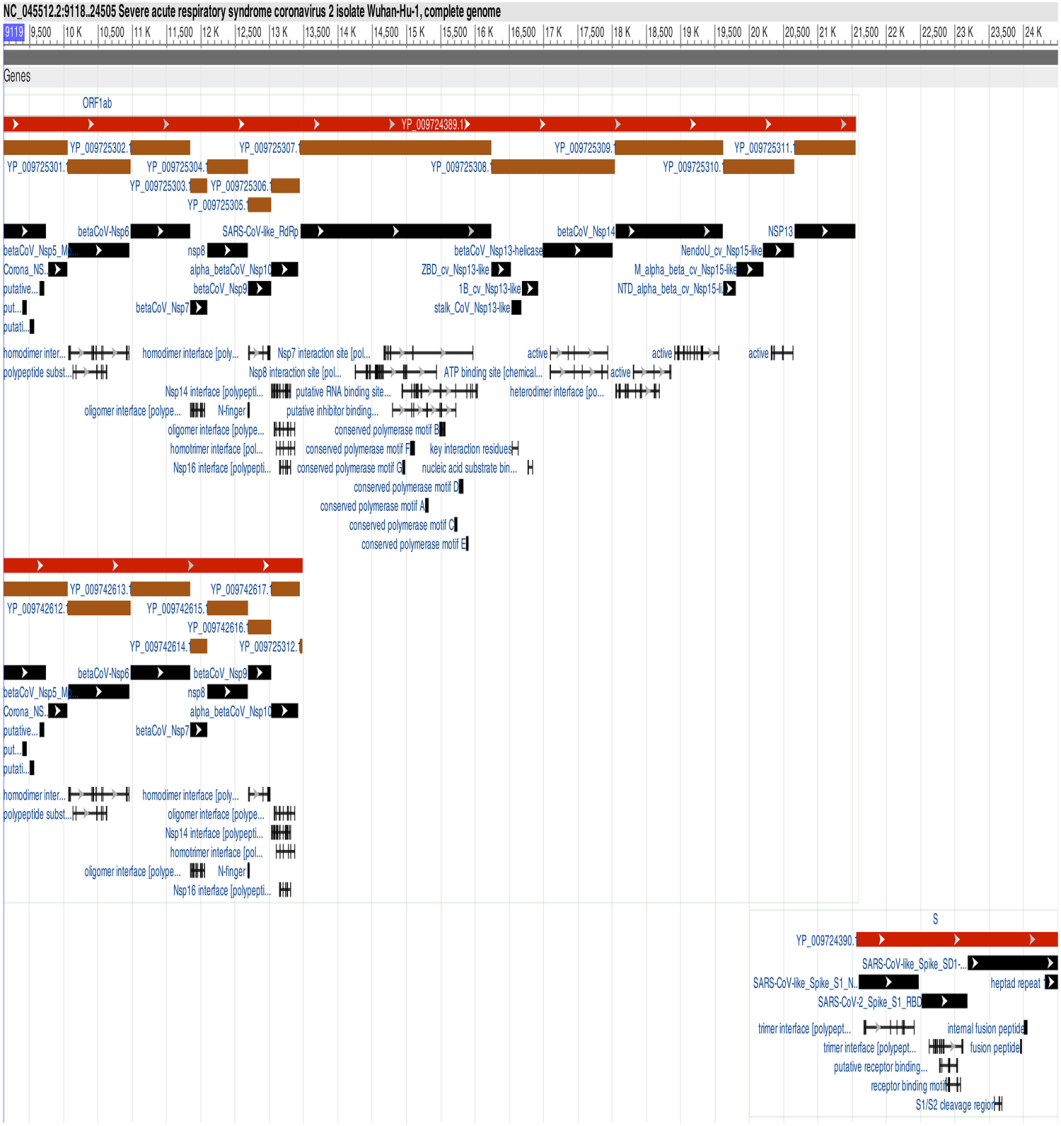
Position on SARS-CoV-2 genome where insertions were detected spanning from *ORF1ab* to start of the *S* gene.

In this study, structural variation is defined as deletion (del) or insertion of at least 50 nucleotides with at least 10 supporting reads. A total of 28 structural variants were identified in 16 of the 49 genome sequences, Table S2 (Supplementary Information). Three-quarters of the structural variants (21/28) were insertions, while the remaining seven were deletions. Ten samples had one insertion each, while six had two insertions, and the remaining six had one deletion each. All insertions were between genomic positions 9119 and 24,505, spanning from *ORF1ab* to the start of the *S* gene, Fig. 1. The longest structural variant was a deletion of 599 base pairs, while the shortest was an insertion of 140 base pairs.

**Figure 2:**
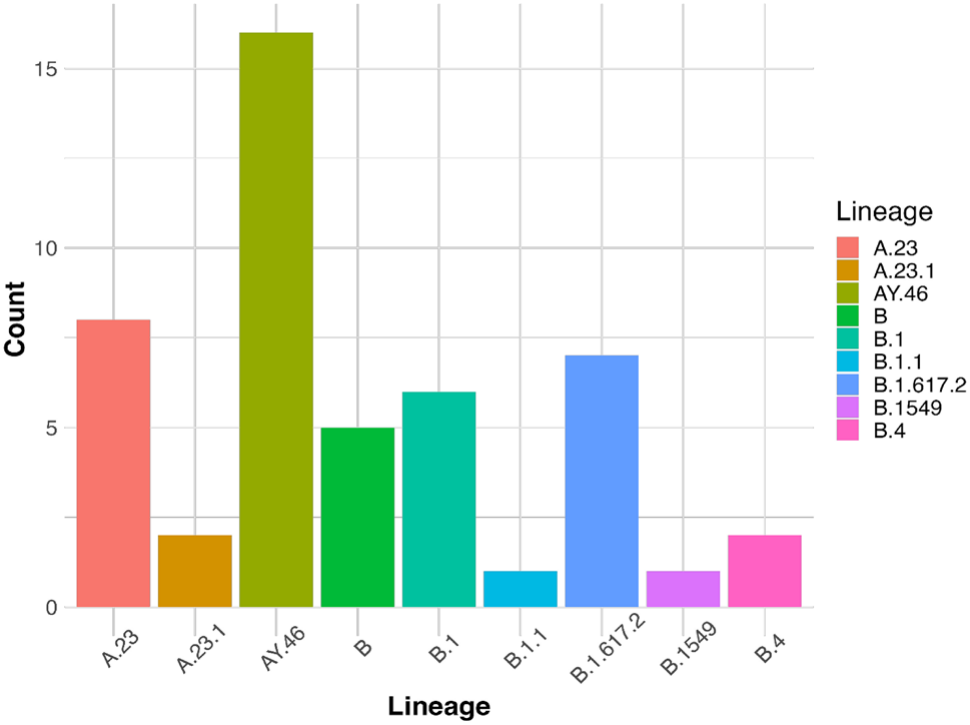
All sequenced SARS-CoV-2 samples belonged to the ‘Delta’ lineage.

Furthermore, when FASTQ sequences were uploaded onto the Next clade programme v2.8.0 (https://clades.nextstrain.org/), we obtained the following SARS-CoV-2 sub-lineages; A.23, A.23.1, AY.46, B.1, B.4, B.1.617.2 and B.1549, all classified as ‘Delta’ lineage. The most prevalent sub-lineages were AY.46 and A.23, Fig. 2. Lineages A.23, A.23.1 and B were identified in samples collected during the 2020 COVID-19 wave while the rest were identified in samples collected during the 2021 COVID-19 wave.

### A comparison of SARS-CoV-2 from Uganda and the rest of East Africa

**Figure 3:**
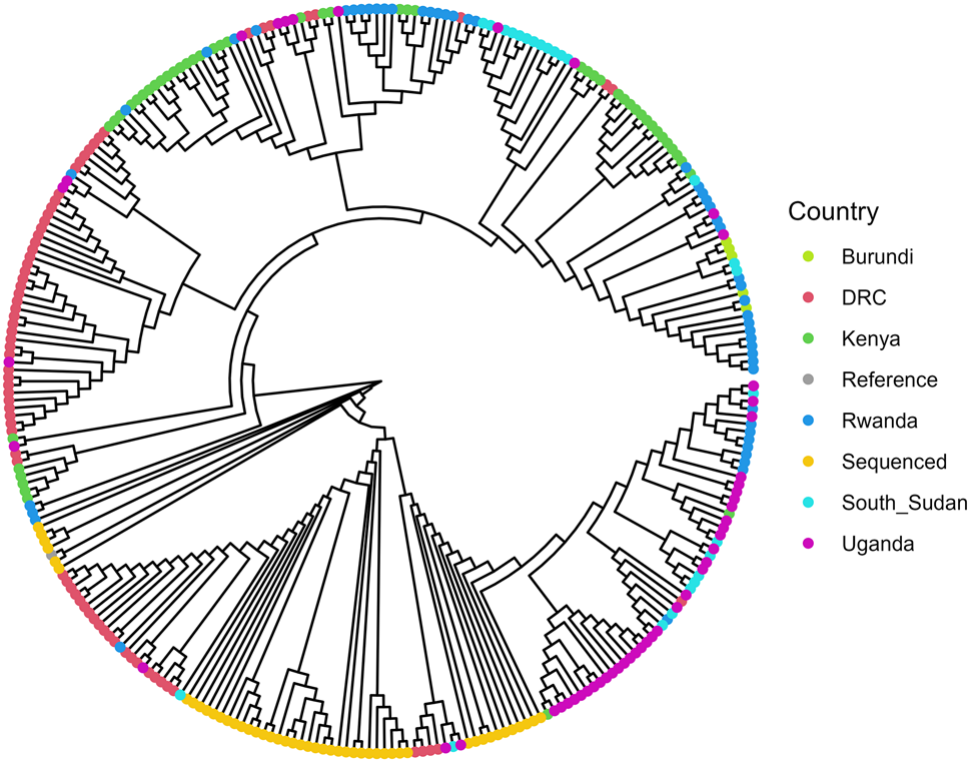
Maximum likelihood phylogenetic tree depicting genetic relatedness between the sequenced SARS-CoV-2 samples in this study (i.e., ‘Sequenced’) and the rest of East Africa. Nodes are *colored* per country. The 49 samples from Uganda clustered and shared a root with other Ugandan sequences from GISAID.

Fifty SARS-CoV-2 sequences from selected Eastern and Central African countries, namely, Uganda, Kenya, Rwanda, and the DRC, as well as 9 and 25 sequences from Burundi and South Sudan, respectively, were obtained from the GISIAD EpiCoV™ website in October, 2022 (https://www.epicov.org/epi3/frontend#254fc1) and analysed together with the sequenced samples from Uganda. Multiple sequence alignment was performed using MAFFT Version 7.310^16^, generating a circular maximum likelihood phylogenetic tree, Fig. 3. The 49 sequences from our study clustered together and shared a root with other Ugandan sequences obtained from GISAID, Fig. 3 and Fig. S1 (Supplementary Information).

## Discussion

In this study, we sequenced and characterized 49 SARS-CoV-2 samples from Uganda, collected between April 2020 and July 2021, using MinION Nanopore sequencing. Overall, the ARTIC protocol used was able to generate the required libraries for successful sequencing on the long-read sequencer. MinION Oxford Nanopore technology enabled the identification of structural variations, which was one of the aims of our study.

We identified 268 unique single nucleotide variants in the 49 genomes, majority of which were in the *ORF1ab*, *S* and *N* genes, which are known mutation hot spots^17, 18^. The *ORF1ab* gene had the highest diversity (144/268) and abundance (705/1456) of mutations. *ORF1ab* is the largest of the SARS-CoV-2 genes, with over 21,000 nucleotides, which increases the probability of mutations^3^. Further, *ORF1ab* has overlapping open reading frames (ORFs) that encode two polyproteins, pp1a and pp1ab, which are cleaved by two viral proteases into 16 nonstructural proteins (nsp1–16). Nonstructural proteins (nsp) include RNA-dependent RNA polymerase (nsp12), exonuclease for proofreading (nsp14), 3ʹ–5ʹ endonuclease (nsp15), RNA binding proteins (nsp9), associated cofactors for replication, papain-like protease, and helicase. The nonstructural proteins allow SARS-CoV-2 viral replication, translation and assembly^19^.

The *S* gene, which codes for the spike protein with which the virus infects the human host via attachment to ACE-2 receptors, was also found to have a high number of mutations. There was a total of 42 unique variants and a total of 333 mutations. The *S* gene is relatively smaller than the *ORF1ab* gene—it is just over 3800 nucleotides^3^. Further, the *S* gene is critical in the evolutionary success of the virus, and thus, mutations in this gene tend not to be tolerated unless they confer some advantage, such as increased infectivity^20, 21^. The most common variants in this gene were 2042C>G, 1841A>G, 1433C>A, 425G>A, 56C>G and 2848G>A, which have all been previously reported and associated with differing severities of the disease; for example, 2042C>G was associated with high viral loads, an increased transmission rate and host immune evasion^12, 19^, while 2848G>A was associated with an increased transmission rate^19^. The SARS-CoV-2 S protein (antigen) directly interacts with the specific host immune cells, and this interaction makes it more susceptible to mutations. This interaction induces a conformational change that directs a formation of endosomes to trigger viral fusion with the host cell under the influence of low pH^22^. For example, we found p.del69/70, p.Glu156Gly, p.Thr95Ile, p.Gly142Asp, p.Glu156Gly, p.Leu452Arg, p.Thr478Lys, and p.Gln493Arg mutations, which are known to decrease sensitivity to neutralizing antibodies and lower binding affinity of the S protein to the ACE2 receptor^19, 23^. Amino acid deletion** (**p.del69/70) has also been reported to be the cause of RT‒PCR failure in the *S* gene^24^.

The *N* gene encodes the N protein, which enables viral assembly in association with envelope proteins. It also has an RNA binding site. The *N* gene is 908 nucleotides long, and we found 24 unique variants and 100 mutations in total. The leading mutation in this gene was 1129G>T. *N* being a major target for diagnostics using the Cepheid Xpert assay and RT‒PCR, such mutations could affect the diagnostic performance of the assays^3, 25^.

The 28 structural variants detected occurred in the *ORF1ab* and *S* gene regions, which are the largest regions on the SARS-CoV-2 genomes. Imposition of these long variations along the genome alters the conformation of the genome, and this have impact on functionality and evolutionary mechanism of viruses^26^. Understanding these conformational alterations lays the groundwork for the creation of agents that interfere with the entry processes^27^.

Furthermore, we used online resources to determine the lineages of the genomes obtained and found the most prevalent sub-lineages to be AY.46 and A.23. Both are considered Delta variants. These sub-lineages were particularly predominant in East Africa https://github.com/cov-lineages/pango-designation/issues/247. Therefore, it is possible that these mutations have arisen locally within East Africa and were being transmitted locally.

A maximum likelihood phylogenetic tree showed all the SARS-CoV-2 detected in our samples clustering together and were more closely related to other Ugandan and DRC SARS-CoV-2 than to those from other neighboring countries. Again, this finding points to the possibility that SARS-CoV-2 infections at the time arose from local spread and were not newly imported. Perhaps, the close relationship between the Ugandan and DRC SARS-CoV-2 samples could also be attributed to the direct human interactions facilitated by cross boarder movements for trade, or conflicts in the DRC that have forced Congolese nationals to migrate into neighboring countries like Uganda; however, these remain speculations until proven by further studies. Moreover, a quantile regression model suggests that globalization, settlement, and population characteristics related to high human mobility and interaction results into SARS-CoV-2 transmission diffusion within or outside a geographical region^28^.

One limitation of our study is that samples were not sequenced with more accurate platforms (e.g., illumina MiSeq) for comparison. Nevertheless, MinION Nanopore sequencing allowed us to characterize SARS-CoV-2 from Uganda—identifying both single nucleotide variants and structural variants in known mutation hot spots of SARS-CoV-2. Importantly, we have shown that SARS-CoV-2 detected in Uganda between April 2020 and July 2021 was a result of infections arising from local spread of the virus.

## Methods

### Study design and setting

This cross-sectional study used 55 stored nasopharyngeal SARS-CoV-2-positive samples and 234 FASTA SARS-CoV-2 genomic sequences collected between April 2020 and July 2021. The study was conducted at the Genomics and Molecular Unit of the Department of Immunology and Molecular Biology at Makerere University College of Health Sciences in Kampala, Uganda. The samples were collected and tested during the COVID-Bank study^29^. Additionally, both Nanopore and Miseq generated SARS-CoV-2 FASTA sequences from East Africa i.e., Uganda, Kenya, Rwanda, Burundi, DRC, and South Sudan were downloaded from GISIAD EpiCoV™, https://www.epicov.org/epi3/frontend#5efc41.

### Viral RNA extraction and amplification

Viral RNA was extracted using a Quick-RNA™ Viral Kit from Zymo Research (USA) following the manufacturer’s guidelines. A multiplex, quantitative Real-Time PCR targeting *N1* and *N2* nucleocapsid genes on the SARS-CoV-2 genome and the human RNase P encoding gene as an internal control, was performed on the extracted RNA using the Luna Universal Probe One-Step RT‒qPCR Kit following the manufacturer’s guidelines (New England Biolabs, NEB, USA).

### cDNA generation, library preparation and MinION sequencing

Upon extraction, viral RNA was first converted to complementary DNA (cDNA) using the ProtoScript® II First Strand cDNA Synthesis Kit (New England Bio labs, NEB, USA) with random primers according to the manufacturer’s instructions. Then, conventional PCR targeting and amplifying the whole SARS-CoV-2 genome using *Artic V3 nCoV-2019* NEBNext ARTIC SARS-CoV-2 Primer Pool A and Pool B (New England Biolabs, NEB, USA) was performed with the Q5® Hot Start High-Fidelity 2× Master Mix according to the manufacturer’s instructions. Library preparation was performed according to the NEBNext ARTIC SARS-CoV-2 Companion kit (ONT). Libraries were normalized, loaded and sequenced on the MK1C flow cell version R9 (Oxford Nanopore Technologies) following the manufacturer’s guidelines.

### Bioinformatics analysis

The raw FAST5 reads were base called, demultiplexed and converted to raw FASTQ reads using MinKNOW and Guppy 5.1.12 + 0a404b92d. The quality of raw FASTQ read files was checked using FASTQC and MultiQC to generate a single quality report for all the samples. The analyses were performed following the bioinformatic workflow described by Bull et al.^26^. To avoid introducing errors, vcf files were generated by filtering at read depth greater than 7 and mapping quality greater than 10 using bcftools, and only SNPs with high quality and a high site depth of coverage were considered in downstream analysis.

### Determination of SNPs and structural variations

Good quality sample reads were aligned to the SARS-CoV-2 reference genome Wuhan-Hu-1 (accession number NC_045512.2) using Minimap2 (2.24-r1122). This generated binary alignment map (BAM) files^30^ which were used in variant calling. Variant calling was performed using Medaka (Medaka haploid variant version 1.7.2) and variant call files (vcf) were generated. Generated variants were annotated using SnpEff version 5.0e. Using BCFtools version 1.8, the resultant annotated variants were filtered at a read depth greater than 7 and a mapping quality greater than 10^31^. To determine structural variations, variant calling and annotation were performed using Sniffles version 2.0.7 and SnpEff version 5.0e, respectively. Structural variants were filtered by excluding variants shorter than 50 bp and having less than 10 support reads^32^.

### Determining genetic relatedness between sequenced SARS-CoV-2 from Uganda and East Africa

Our sample sequences and SARS-CoV-2 FASTA sequences in the GISAID database from Uganda, Kenya, Rwanda, Burundi, DRC and South Sudan were used. The 49 FASTQ sample reads were assembled using the Flye version 2.9.1-b1780 set with ONT regular reads of < 20% error (–nanoraw), with 5 polishing iterations, and scaffolding using a graph excluding contigs representing alternative haplotypes (–no-alt-contigs). The resultant assembled contigs were joined using contigMerger to generate a single scaffold per sample. The per sample scaffold was combined into a single multifasta file that was used in the phylogenetic analysis. A total of 7221 FASTA sequences were downloaded from GISIAD EpiCoV™ between April 2020 and 2021; 834 from Uganda, 565 from Rwanda, 9 from Burundi, 80 from South Sudan, 766 from DRC and 4968 from Kenya. These were merged into a single multifasta file, and poor-quality sequences having any ambiguous bases (N) were excluded using the biopython package. Multiple sequence alignment was performed using MAFFT Version 7.310, and the phylogenetic tree was constructed using the maximum likelihood method in MEGA version 11. The resultant tree generated was reported into R version 4.2.1 and manipulated using the ggtree package. https://www.molecularecologist.com/2017/02/08/phylogenetic-trees-in-r-using-ggtree/.

### Ethical considerations

Approval to conduct the study was received from the Makerere University School of Biomedical Sciences Research and Ethics Committee (SBS-REC 2022-124). As well, approval to use archived samples was obtained from the Department of Immunology and Molecular biology, Makerere University, College of Health Sciences. All procedures described were performed in accordance with relevant national/international guidelines/regulations; informed consent was obtained from the participants and/or their legal guardians in whom samples for SARS-CoV-2 testing were obtained.

### Supplementary Information

Supplementary Information.

## Data Availability

The datasets generated and/or analysed during the current study are available in the GISAID https://gisaid.org/ repository; the raw sequence data generated in this study was deposited in the NCBI BioProject database https://www.ncbi.nlm.nih.gov/bioproject/922477, accession number PRJNA922477.
