# Supplementary material for: Genomic characterization of SARS-CoV-2 from Uganda using MinION nanopore sequencing
Source: Sci Rep. 2023 Nov 22;13:20507. doi: 10.1038/s41598-023-47379-z (PMC10665338; doi:10.1038/s41598-023-47379-z)
Supplement: Supplementary file 1 — Supplementary Information. [file 41598_2023_47379_MOESM1_ESM.docx]

Genomic characterization of SARS-CoV-2 from Uganda using MinION Nanopore Sequencing

Praiscillia Kia^1*^, Eric Katagirya^1^, Fredrick Elishama Kakembo^2^, Doreen Ato Adera^3^, Moses Luutu Nsubuga^1^, Fahim Yiga^1^, Sharley Melissa Aloyo ^1^, Brendah Ronah Aujat^1^, Denis Foe Anguyo^4^, Fred Ashaba Katabazi^1^, Edgar Kigozi^1^, Moses L Joloba^1^, and David Patrick Kateete^1*^

Supplementary information:

**Table S1. Mutations detected (n=49 SARS-CoV-2 genome sequences)**

| Freq | Position | Depth | Mutation | Gene | Nucleotide Variation | Amino acid Change |
| --- | --- | --- | --- | --- | --- | --- |
| 41 | 23604 | 2737 | missense_variant | *S* | c.2042C>G | p.Pro681Arg |
| 33 | 23403 | 1135 | missense_variant | *S* | c.1841A>G | p.Asp614Gly |
| 26 | 22995 | 100 | missense_variant | *S* | c.1433C>A | p.Thr478Lys |
| 25 | 21987 | 119 | missense_variant | *S* | c.425G>A | p.Gly142Asp |
| 25 | 22028 | 118 | disruptive_inframe_deletion | *S* | c.467_472delAGTTCA | p.Glu156_Arg158delinsGly |
| 24 | 21618 | 227 | missense_variant | *S* | c.56C>G | p.Thr19Arg |
| 23 | 24410 | 7979 | missense_variant | *S* | c.2848G>A | p.Asp950Asn |
| 21 | 22917 | 97 | missense_variant | *S* | c.1355T>G | p.Leu452Arg |
| 12 | 22000 | 2960 | synonymous_variant | *S* | c.438C>T | p.His146His |
| 11 | 22033 | 2992 | missense_variant | *S* | c.471C>A | p.Phe157Leu |
| 9 | 22661 | 4866 | missense_variant | *S* | c.1099G>T | p.Val367Phe |
| 9 | 23075 | 98 | missense_variant | *S* | c.1513T>C | p.Tyr505His |
| 9 | 23401 | 1855 | missense_variant | *S* | c.1839G>T | p.Gln613His |
| 9 | 24097 | 7881 | synonymous_variant | *S* | c.2535T>C | p.Ala845Ala |
| 8 | 23202 | 444 | missense_variant | *S* | c.1640C>A | p.Thr547Lys |
| 6 | 23055 | 66 | missense_variant | *S* | c.1493A>G | p.Gln498Arg |
| 6 | 24424 | 2132 | missense_variant | *S* | c.2862A>T | p.Gln954His |
| 3 | 23048 | 98 | missense_variant | *S* | c.1486G>A | p.Gly496Ser |
| 3 | 24503 | 2121 | missense_variant | *S* | c.2941C>T | p.Leu981Phe |
| 2 | 21624 | 145 | missense_variant | *S* | c.62G>T | p.Arg21Ile |
| 2 | 21867 | 414 | missense_variant | *S* | c.305G>T | p.Arg102Ile |
| 2 | 22203 | 31 | missense_variant | *S* | c.641G>T | p.Arg214Leu |
| 2 | 23013 | 111 | missense_variant | *S* | c.1451A>C | p.Glu484Ala |
| 2 | 23040 | 113 | missense_variant | *S* | c.1478A>G | p.Gln493Arg |
| 2 | 23063 | 106 | missense_variant | *S* | c.1501A>T | p.Asn501Tyr |
| 2 | 24469 | 2130 | missense_variant | *S* | c.2907T>A | p.Asn969Lys |
| 1 | 21764 | 14 | disruptive_inframe_deletion | *S* | c.204_209delACATGT | p.His69_Val70del |
| 1 | 21765 | 49 | frameshift_variant | *S* | c.204_208delACATG | p.His69fs |
| 1 | 21846 | 13 | missense_variant | *S* | c.284C>T | p.Thr95Ile |
| 1 | 21979 | 11 | frameshift_variant | *S* | c.422dupT | p.Leu141fs |
| 1 | 21997 | 7509 | synonymous_variant | *S* | c.435C>T | p.Tyr145Tyr |
| 1 | 22963 | 1125 | synonymous_variant | *S* | c.1401T>C | p.Asp467Asp |
| 1 | 23116 | 8006 | frameshift_variant | *S* | c.1554_1555insG | p.His519fs |
| 1 | 23122 | 8011 | disruptive_inframe_insertion | *S* | c.1562_1563insTCC | p.Pro521dup |
| 1 | 23311 | 298 | missense_variant | *S* | c.1749_1750delGAinsTC | p.GluIle583AspLeu |
| 1 | 23593 | 2997 | missense_variant | *S* | c.2031G>T | p.Gln677His |
| 1 | 8026 | 8026 | missense_variant | *S* | c.2042C>G | p.Pro681Arg |
| 1 | 23698 | 634 | synonymous_variant | *S* | c.2136T>A | p.Ile712Ile |
| 1 | 23932 | 1441 | synonymous_variant | *S* | c.2370A>G | p.Lys790Lys |
| 1 | 24095 | 7994 | missense_variant | *S* | c.2533G>T | p.Ala845Ser |
| 1 | 24992 | 3286 | missense_variant | *S* | c.3430G>C | p.Glu1144Gln |
| 1 | 25357 | 34 | synonymous_variant | *S* | c.3795C>G | p.Leu1265Leu |
| 21 | 28271 | 58 | intergenic_region | *ORF8-N* | n.28271A>T |  |
| 7 | 28270 | 221 | intergenic_region | *ORF8-N* | n.28271delA |  |
| 11 | 28247 | 213 | conservative_inframe_deletion | *ORF8* | c.355_360delGATTTC | p.Asp119_Phe120del |
| 4 | 28144 | 109 | missense_variant | *ORF8* | c.251T>C | p.Leu84Ser |
| 3 | 28167 | 99 | missense_variant | *ORF8* | c.274G>A | p.Glu92Lys |
| 1 | 28076 | 2803 | frameshift_variant | *ORF8* | c.183_184insAG | p.Val62fs |
| 18 | 27874 | 214 | missense_variant | *ORF7b* | c.119C>T | p.Thr40Ile |
| 32 | 27638 | 1455 | missense_variant | *ORF7a* | c.245T>C | p.Val82Ala |
| 23 | 27752 | 176 | missense_variant | *ORF7a* | c.359C>T | p.Thr120Ile |
| 1 | 27520 | 1506 | missense_variant | *ORF7a* | c.127A>G | p.Asn43Asp |
| 4 | 27259 | 377 | synonymous_variant | *ORF6* | c.58A>C | p.Arg20Arg |
| 3 | 27297 | 248 | frameshift_variant | *ORF6* | c.96_97insT | p.Ile33fs |
| 2 | 27295 | 251 | missense_variant | *ORF6* | c.94A>G | p.Ile32Val |
| 23 | 25469 | 32 | missense_variant | *ORF3a* | c.77C>T | p.Ser26Leu |
| 1 | 25482 | 14 | synonymous_variant | *ORF3a* | c.90C>T | p.Arg30Arg |
| 1 | 25521 | 431 | synonymous_variant | *ORF3a* | c.129C>T | p.Phe43Phe |
| 1 | 25729 | 8030 | missense_variant | *ORF3a* | c.337T>C | p.Tyr113His |
| 1 | 26001 | 8034 | synonymous_variant | *ORF3a* | c.609A>G | p.Leu203Leu |
| 1 | 26162 | 146 | missense_variant | *ORF3a* | c.770A>G | p.Asn257Ser |
| 36 | 14408 | 1743 | synonymous_variant | *ORF1ab* | c.14143C>T | p.Leu4715Leu |
| 34 | 3037 | 577 | synonymous_variant | *ORF1ab* | c.2772C>T | p.Phe924Phe |
| 34 | 10029 | 1851 | missense_variant | *ORF1ab* | c.9764C>T | p.Thr3255Ile |
| 30 | 19220 | 438 | synonymous_variant | *ORF1ab* | c.18955C>T | p.Leu6319Leu |
| 29 | 17259 | 265 | missense_variant | *ORF1ab* | c.16994G>T | p.Ser5665Ile |
| 29 | 20486 | 5939 | missense_variant | *ORF1ab* | c.20221A>G | p.Ser6741Gly |
| 27 | 4181 | 4015 | missense_variant | *ORF1ab* | c.3916G>T | p.Ala1306Ser |
| 27 | 8986 | 5848 | synonymous_variant | *ORF1ab* | c.8721C>T | p.Asp2907Asp |
| 24 | 7124 | 4843 | missense_variant | *ORF1ab* | c.6859C>T | p.Pro2287Ser |
| 23 | 9053 | 5907 | missense_variant | *ORF1ab* | c.8788G>T | p.Val2930Leu |
| 21 | 10977 | 2686 | missense_variant | *ORF1ab* | c.10712C>T | p.Ala3571Val |
| 21 | 11332 | 38 | synonymous_variant | *ORF1ab* | c.11067A>G | p.Val3689Val |
| 20 | 16466 | 3427 | missense_variant | *ORF1ab* | c.16201C>T | p.His5401Tyr |
| 19 | 415 | 27 | synonymous_variant | *ORF1ab* | c.150T>C | p.Thr50Thr |
| 19 | 11201 | 2682 | missense_variant | *ORF1ab* | c.10936A>G | p.Thr3646Ala |
| 17 | 13195 | 566 | synonymous_variant | *ORF1ab* | c.12930T>C | p.Val4310Val |
| 16 | 14033 | 324 | frameshift_variant&missense_variant | *ORF1ab* | c.13768delAinsGG | p.Met4590fs |
| 14 | 15451 | 6238 | synonymous_variant | *ORF1ab* | c.15186G>A | p.Ala5062Ala |
| 12 | 4573 | 13 | synonymous_variant | *ORF1ab* | c.4308C>T | p.Asn1436Asn |
| 11 | 10423 | 6164 | synonymous_variant | *ORF1ab* | c.10158T>A | p.Ser3386Ser |
| 10 | 5386 | 1270 | synonymous_variant | *ORF1ab* | c.5121T>G | p.Ala1707Ala |
| 10 | 8782 | 8057 | synonymous_variant | *ORF1ab* | c.8517C>T | p.Ser2839Ser |
| 10 | 9246 | 3270 | missense_variant | *ORF1ab* | c.8981C>G | p.Ala2994Gly |
| 10 | 9249 | 3237 | missense_variant | *ORF1ab* | c.8984G>C | p.Gly2995Ala |
| 10 | 10747 | 5456 | synonymous_variant | *ORF1ab* | c.10482C>T | p.Asn3494Asn |
| 10 | 11303 | 46 | disruptive_inframe_deletion | *ORF1ab* | c.11040_11042delAGA | p.Lys3680_Asp3681delinsAsn |
| 10 | 16575 | 7342 | missense_variant | *ORF1ab* | c.16310C>T | p.Thr5437Ile |
| 10 | 17745 | 221 | missense_variant | *ORF1ab* | c.17480C>T | p.Thr5827Ile |
| 9 | 11230 | 3298 | missense_variant | *ORF1ab* | c.10965G>T | p.Met3655Ile |
| 5 | 18163 | 14 | synonymous_variant | *ORF1ab* | c.17898A>G | p.Thr5966Thr |
| 5 | 18877 | 1285 | frameshift_variant&stop_lost&synonymous_variant | *ORF1ab* | c.18612delCinsGG | p.Ter6205fs |
| 4 | 1191 | 3895 | missense_variant | *ORF1ab* | c.926C>T | p.Pro309Leu |
| 4 | 13459 | 25 | frameshift_variant | *ORF1ab* | c.13195_13199delTTTTT | p.Phe4399fs |
| 4 | 14560 | 18 | missense_variant | *ORF1ab* | c.14295_14296delTAinsCT | p.Met4766Leu |
| 3 | 4657 | 905 | synonymous_variant | *ORF1ab* | c.4392G>A | p.Arg1464Arg |
| 3 | 13369 | 69 | frameshift_variant | *ORF1ab* | c.13106_13212del | p.Val4369fs |
| 3 | 20489 | 5789 | frameshift_variant | *ORF1ab* | c.20224_20225insC | p.Val6742fs |
| 2 | 3814 | 51 | frameshift_variant&stop_gained | *ORF1ab* | c.3549_3550delTGinsA | p.Tyr1183fs |
| 2 | 6402 | 19 | missense_variant | *ORF1ab* | c.6137C>T | p.Pro2046Leu |
| 2 | 7575 | 2306 | frameshift_variant&stop_gained | *ORF1ab* | c.7310_7311insGG | p.Tyr2437fs |
| 2 | 7580 | 1161 | missense_variant | *ORF1ab* | c.7315A>G | p.Asn2439Asp |
| 2 | 11052 | 2143 | missense_variant | *ORF1ab* | c.10787A>G | p.Gln3596Arg |
| 2 | 11266 | 2201 | missense_variant | *ORF1ab* | c.11001G>T | p.Leu3667Phe |
| 2 | 13006 | 82 | frameshift_variant | *ORF1ab* | c.12742delG | p.Ala4248fs |
| 2 | 13373 | 27 | disruptive_inframe_deletion | *ORF1ab* | c.13109_13213del | p.Cys4370_Val4405delinsLeu |
| 2 | 13453 | 70 | frameshift_variant | *ORF1ab* | c.13190_13193delAATC | p.Gln4397fs |
| 2 | 17067 | 174 | missense_variant | *ORF1ab* | c.16802T>C | p.Ile5601Thr |
| 2 | 18060 | 60 | missense_variant | *ORF1ab* | c.17795C>T | p.Ser5932Phe |
| 2 | 18176 | 54 | synonymous_variant | *ORF1ab* | c.17911C>T | p.Leu5971Leu |
| 2 | 18655 | 1726 | synonymous_variant | *ORF1ab* | c.18390A>T | p.Ala6130Ala |
| 2 | 20492 | 640 | missense_variant | *ORF1ab* | c.20227T>C | p.Cys6743Arg |
| 2 | 21270 | 2363 | missense_variant | *ORF1ab* | c.21005A>G | p.His7002Arg |
| 1 | 507 | 756 | frameshift_variant | *ORF1ab* | c.243_244delTG | p.His81fs |
| 1 | 509 | 505 | missense_variant | *ORF1ab* | c.244G>T | p.Gly82Cys |
| 1 | 515 | 21 | disruptive_inframe_deletion | *ORF1ab* | c.252_254delTAT | p.Met85del |
| 1 | 544 | 99 | missense_variant | *ORF1ab* | c.279A>T | p.Glu93Asp |
| 1 | 576 | 59 | disruptive_inframe_deletion | *ORF1ab* | c.317_397delTCCTTGTCCCTCATGTGGGCGAAATACCAGTGGCTTACCGCAAGGTTCTTCTTCGTAAGAACGGTAATAAAGGAGCTGGTG | p.Val106_Gly132del |
| 1 | 711 | 25 | missense_variant | *ORF1ab* | c.446T>G | p.Leu149Arg |
| 1 | 793 | 21 | frameshift_variant | *ORF1ab* | c.530_534delTTAAC | p.Leu177fs |
| 1 | 21 | 21 | frameshift_variant | *ORF1ab* | c.530_534delTTAAC | p.Leu177fs |
| 1 | 806 | 21 | frameshift_variant | *ORF1ab* | c.542_555delCATACACTCGCTAT | p.Ala181fs |
| 1 | 823 | 120 | synonymous_variant | *ORF1ab* | c.558C>T | p.Val186Val |
| 1 | 824 | 21 | frameshift_variant | *ORF1ab* | c.560_563delATAA | p.Asp187fs |
| 1 | 829 | 22 | frameshift_variant | *ORF1ab* | c.565_566delAA | p.Asn189fs |
| 1 | 925 | 2927 | synonymous_variant | *ORF1ab* | c.660C>T | p.Asp220Asp |
| 1 | 1346 | 8002 | missense_variant | *ORF1ab* | c.1081C>T | p.Pro361Ser |
| 1 | 2695 | 667 | synonymous_variant | *ORF1ab* | c.2430C>T | p.Asn810Asn |
| 1 | 2832 | 33 | missense_variant | *ORF1ab* | c.2567A>G | p.Lys856Arg |
| 1 | 3300 | 205 | stop_gained | *ORF1ab* | c.3035T>G | p.Leu1012* |
| 1 | 3367 | 338 | synonymous_variant | *ORF1ab* | c.3102T>C | p.Leu1034Leu |
| 1 | 3492 | 716 | frameshift_variant | *ORF1ab* | c.3228delT | p.Asn1076fs |
| 1 | 3497 | 25 | frameshift_variant | *ORF1ab* | c.3233_3234delCT | p.Ala1078fs |
| 1 | 4331 | 240 | synonymous_variant | *ORF1ab* | c.4066C>T | p.Leu1356Leu |
| 1 | 4999 | 7285 | synonymous_variant | *ORF1ab* | c.4734C>T | p.Asn1578Asn |
| 1 | 5935 | 215 | synonymous_variant | *ORF1ab* | c.5670A>G | p.Thr1890Thr |
| 1 | 6568 | 12 | synonymous_variant | *ORF1ab* | c.6303C>T | p.Asp2101Asp |
| 1 | 6628 | 42 | synonymous_variant | *ORF1ab* | c.6363C>T | p.Thr2121Thr |
| 1 | 7581 | 132 | missense_variant | *ORF1ab* | c.7316A>G | p.Asn2439Ser |
| 1 | 7583 | 1152 | frameshift_variant&missense_variant | *ORF1ab* | c.7318delGinsCA | p.Gly2440fs |
| 1 | 8326 | 7109 | synonymous_variant | *ORF1ab* | c.8061C>T | p.Asp2687Asp |
| 1 | 8935 | 5868 | synonymous_variant | *ORF1ab* | c.8670T>C | p.Ser2890Ser |
| 1 | 9244 | 5440 | disruptive_inframe_insertion | *ORF1ab* | c.8980_8981insGTG | p.Glu2993_Ala2994insGly |
| 1 | 9250 | 3603 | synonymous_variant | *ORF1ab* | c.8985T>A | p.Gly2995Gly |
| 1 | 9251 | 1202 | frameshift_variant | *ORF1ab* | c.8986_8987insC | p.Val2996fs |
| 1 | 9252 | 3589 | frameshift_variant | *ORF1ab* | c.8987_8988insC | p.Cys2997fs |
| 1 | 9278 | 2368 | missense_variant | *ORF1ab* | c.9013G>A | p.Val3005Ile |
| 1 | 9955 | 5655 | synonymous_variant | *ORF1ab* | c.9690T>G | p.Ala3230Ala |
| 1 | 11121 | 130 | frameshift_variant | *ORF1ab* | c.10857_10858delTG | p.Ala3620fs |
| 1 | 11126 | 131 | missense_variant | *ORF1ab* | c.10861A>T | p.Met3621Leu |
| 1 | 11128 | 131 | missense_variant | *ORF1ab* | c.10863G>C | p.Met3621Ile |
| 1 | 11130 | 133 | missense_variant | *ORF1ab* | c.10865C>G | p.Ser3622Cys |
| 1 | 11276 | 12 | disruptive_inframe_deletion | *ORF1ab* | c.11012_11014delATA | p.Asp3671_Thr3672delinsAla |
| 1 | 11413 | 2103 | synonymous_variant | *ORF1ab* | c.11148A>G | p.Thr3716Thr |
| 1 | 11514 | 8015 | missense_variant | *ORF1ab* | c.11249C>T | p.Thr3750Ile |
| 1 | 12171 | 4388 | missense_variant | *ORF1ab* | c.11906C>T | p.Ala3969Val |
| 1 | 12393 | 933 | missense_variant | *ORF1ab* | c.12128A>G | p.Asp4043Gly |
| 1 | 12789 | 701 | missense_variant | *ORF1ab* | c.12524C>T | p.Thr4175Ile |
| 1 | 13372 | 14 | conservative_inframe_deletion | *ORF1ab* | c.13108_13212del | p.Cys4370_Ala4404del |
| 1 | 13376 | 39 | frameshift_variant | *ORF1ab* | c.13113_13119delCGTCTGC | p.Met4375fs |
| 1 | 13384 | 285 | disruptive_inframe_deletion | *ORF1ab* | c.13122_13139delTATGTGGAAAGGTTATGG | p.Met4375_Gly4380del |
| 1 | 13388 | 18 | frameshift_variant | *ORF1ab* | c.13124_13193delTGTGGAAAGGTTATGGCTGTAGTTGTGATCAACTCCGCGAACCCATGCTTCAGTCAGCTGATGCACAATC | p.Met4375fs |
| 1 | 13397 | 38 | frameshift_variant | *ORF1ab* | c.13134_13138delTTATG | p.Tyr4379fs |
| 1 | 13404 | 38 | frameshift_variant | *ORF1ab* | c.13141_13174delTGTAGTTGTGATCAACTCCGCGAACCCATGCTTC | p.Cys4381fs |
| 1 | 13405 | 278 | frameshift_variant&missense_variant | *ORF1ab* | c.13140_13159delCTGTAGTTGTGATCAACTCCinsGGG | p.Cys4381fs |
| 1 | 13410 | 27 | frameshift_variant | *ORF1ab* | c.13146_13147delTT | p.Ser4382fs |
| 1 | 13425 | 278 | frameshift_variant | *ORF1ab* | c.13161_13189delCGAACCCATGCTTCAGTCAGCTGATGCAC | p.Glu4388fs |
| 1 | 13432 | 70 | frameshift_variant | *ORF1ab* | c.13168_13169delAT | p.Met4390fs |
| 1 | 13435 | 25 | disruptive_inframe_deletion | *ORF1ab* | c.13172_13174delTTC | p.Leu4391del |
| 1 | 13436 | 70 | frameshift_variant | *ORF1ab* | c.13172_13173delTT | p.Leu4391fs |
| 1 | 13443 | 38 | frameshift_variant | *ORF1ab* | c.13179_13188delAGCTGATGCA | p.Ala4394fs |
| 1 | 13445 | 27 | frameshift_variant | *ORF1ab* | c.13181_13182delCT | p.Ala4394fs |
| 1 | 13452 | 42 | frameshift_variant | *ORF1ab* | c.13188_13213delACAATCGTTTTTAAACGGGTTTGCGG | p.Gln4397fs |
| 1 | 13455 | 25 | disruptive_inframe_deletion | *ORF1ab* | c.13191_13193delATC | p.Ser4398del |
| 1 | 13457 | 18 | conservative_inframe_deletion | *ORF1ab* | c.13195_13212delTTTTTAAACGGGTTTGCG | p.Phe4399_Ala4404del |
| 1 | 13465 | 25 | conservative_inframe_deletion | *ORF1ab* | c.13201_13203delAAC | p.Asn4401del |
| 1 | 13470 | 27 | conservative_inframe_deletion | *ORF1ab* | c.13207_13212delTTTGCG | p.Phe4403_Ala4404del |
| 1 | 13471 | 32 | conservative_inframe_deletion | *ORF1ab* | c.13207_13209delTTT | p.Phe4403del |
| 1 | 13730 | 7157 | synonymous_variant | *ORF1ab* | c.13465C>T | p.Leu4489Leu |
| 1 | 14041 | 77 | synonymous_variant | *ORF1ab* | c.13776A>T | p.Val4592Val |
| 1 | 14874 | 209 | missense_variant | *ORF1ab* | c.14609G>T | p.Ser4870Ile |
| 1 | 15199 | 2593 | synonymous_variant | *ORF1ab* | c.14934G>T | p.Leu4978Leu |
| 1 | 15569 | 8082 | synonymous_variant | *ORF1ab* | c.15304C>T | p.Leu5102Leu |
| 1 | 15907 | 8026 | synonymous_variant | *ORF1ab* | c.15642G>A | p.Arg5214Arg |
| 1 | 16034 | 379 | missense_variant | *ORF1ab* | c.15769A>G | p.Met5257Val |
| 1 | 16236 | 1625 | missense_variant | *ORF1ab* | c.15971G>T | p.Arg5324Met |
| 1 | 7579 | 7579 | missense_variant | *ORF1ab* | c.16201C>T | p.His5401Tyr |
| 1 | 16692 | 6199 | missense_variant | *ORF1ab* | c.16427T>C | p.Leu5476Pro |
| 1 | 16877 | 79 | stop_gained | *ORF1ab* | c.16612C>T | p.Gln5538* |
| 1 | 17518 | 2671 | synonymous_variant | *ORF1ab* | c.17253C>T | p.Asp5751Asp |
| 1 | 17643 | 77 | missense_variant | *ORF1ab* | c.17378T>C | p.Leu5793Pro |
| 1 | 18266 | 13 | missense_variant | *ORF1ab* | c.18001_18002delGCinsAT | p.Ala6001Met |
| 1 | 18647 | 7067 | synonymous_variant | *ORF1ab* | c.18382C>T | p.Leu6128Leu |
| 1 | 18878 | 1277 | frameshift_variant&stop_lost | *ORF1ab* | c.18614delA | p.Ter6205fs |
| 1 | 18882 | 387 | frameshift_variant | *ORF1ab* | c.18618dupG | p.Ser6207fs |
| 1 | 18885 | 1265 | frameshift_variant | *ORF1ab* | c.18620_18621insA | p.Thr6208fs |
| 1 | 19269 | 491 | missense_variant | *ORF1ab* | c.19004C>T | p.Thr6335Ile |
| 1 | 19275 | 25 | missense_variant | *ORF1ab* | c.19010T>A | p.Leu6337Gln |
| 1 | 19602 | 1060 | missense_variant | *ORF1ab* | c.19337C>T | p.Thr6446Ile |
| 1 | 19669 | 1059 | frameshift_variant&stop_gained | *ORF1ab* | c.19404_19405insAGGTGCT | p.Asp6469fs |
| 1 | 8020 | 8020 | synonymous_variant | *ORF1ab* | c.20136T>C | p.Asn6712Asn |
| 1 | 20401 | 8020 | synonymous_variant | *ORF1ab* | c.20136T>C | p.Asn6712Asn |
| 1 | 20494 | 1446 | missense_variant | *ORF1ab* | c.20229T>G | p.Cys6743Trp |
| 1 | 20707 | 323 | missense_variant | *ORF1ab* | c.20442A>C | p.Lys6814Asn |
| 1 | 21048 | 349 | missense_variant | *ORF1ab* | c.20783T>C | p.Ile6928Thr |
| 37 | 29742 | 7712 | intergenic_region | ORF10-CHR_END | n.29742G>T |  |
| 3 | 29842 | 15 | intergenic_region | ORF10-CHR_END | n.29842_29843insC |  |
| 3 | 29850 | 13 | intergenic_region | ORF10-CHR_END | n.29850_29851insCCT |  |
| 2 | 29836 | 13 | intergenic_region | ORF10-CHR_END |  |  |
| 2 | 29844 | 23 | intergenic_region | ORF10-CHR_END | n.29844_29845insCTCG |  |
| 2 | 29846 | 13 | intergenic_region | ORF10-CHR_END | n.29846_29847insGACG |  |
| 1 | 29727 | 1091 | intergenic_region | ORF10-CHR_END | n.29728_29744delTTCACCGAGGCCACGCG |  |
| 1 | 29729 | 2216 | intergenic_region | ORF10-CHR_END | n.29730_29733delCACC |  |
| 1 | 29733 | 4898 | intergenic_region | ORF10-CHR_END | n.29733C>T |  |
| 1 | 29749 | 623 | intergenic_region | ORF10-CHR_END | n.29750_29753delCGAT |  |
| 1 | 29751 | 7899 | intergenic_region | ORF10-CHR_END | n.29751G>C |  |
| 1 | 29781 | 1387 | intergenic_region | ORF10-CHR_END | n.29781G>T |  |
| 1 | 29834 | 32 | intergenic_region | ORF10-CHR_END | n.29834_29835insGGG |  |
| 1 | 29835 | 13 | intergenic_region | ORF10-CHR_END |  |  |
| 1 | 29839 | 13 | intergenic_region | ORF10-CHR_END | n.29839_29840insC |  |
| 1 | 29843 | 13 | intergenic_region | ORF10-CHR_END | n.29843G>C |  |
| 1 | 29852 | 15 | intergenic_region | ORF10-CHR_END | n.29852_29853insGTC |  |
| 1 | 29854 | 13 | intergenic_region | ORF10-CHR_END | n.29854_29855insAA |  |
| 1 | 29856 | 16 | intergenic_region | ORF10-CHR_END | n.29856_29857insTAA |  |
| 1 | 29859 | 16 | intergenic_region | ORF10-CHR_END | n.29859_29860insAATA |  |
| 1 | 29860 | 15 | intergenic_region | ORF10-CHR_END | n.29860_29861insACT |  |
| 1 | 29862 | 12 | intergenic_region | ORF10-CHR_END | n.29862G>A |  |
| 2 | 29543 | 1138 | intergenic_region | N-ORF10 | n.29543G>T |  |
| 25 | 29402 | 7985 | missense_variant | *N* | c.1129G>T | p.Asp377Tyr |
| 12 | 28311 | 58 | missense_variant | *N* | c.38C>T | p.Pro13Leu |
| 12 | 28361 | 54 | disruptive_inframe_deletion | *N* | c.90_98delAGAACGCAG | p.Glu31_Ser33del |
| 8 | 28881 | 19 | missense_variant | *N* | c.608G>T | p.Arg203Met |
| 8 | 28916 | 17 | missense_variant | *N* | c.643G>T | p.Gly215Cys |
| 6 | 28364 | 114 | disruptive_inframe_deletion | *N* | c.92_97delAACGCA | p.Glu31_Ser33delinsGly |
| 6 | 28461 | 18 | missense_variant | *N* | c.188A>G | p.Asp63Gly |
| 4 | 28626 | 124 | missense_variant | *N* | c.353A>C | p.Glu118Ala |
| 3 | 28378 | 1572 | synonymous_variant | *N* | c.105G>C | p.Ala35Ala |
| 2 | 28764 | 156 | missense_variant | *N* | c.491G>A | p.Gly164Glu |
| 1 | 28362 | 230 | frameshift_variant | *N* | c.92_98delAACGCAG | p.Glu31fs |
| 1 | 28367 | 296 | disruptive_inframe_deletion | *N* | c.96_98delCAG | p.Ser33del |
| 1 | 28614 | 112 | missense_variant | *N* | c.341G>A | p.Gly114Glu |
| 1 | 28616 | 112 | missense_variant | *N* | c.343A>C | p.Thr115Pro |
| 1 | 28620 | 114 | missense_variant | *N* | c.347G>T | p.Gly116Val |
| 1 | 28621 | 60 | synonymous_variant | *N* | c.348G>A | p.Gly116Gly |
| 1 | 28622 | 115 | frameshift_variant | *N* | c.350_351dupCA | p.Glu118fs |
| 1 | 28657 | 12 | synonymous_variant | *N* | c.384C>T | p.Asp128Asp |
| 1 | 28713 | 699 | frameshift_variant | *N* | c.442_443delAC | p.Thr148fs |
| 1 | 29133 | 2260 | missense_variant | *N* | c.860G>T | p.Gly287Val |
| 1 | 29167 | 512 | frameshift_variant | *N* | c.897delA | p.Lys299fs |
| 1 | 29225 | 13 | frameshift_variant | *N* | c.954_976delGCGCATTGGCATGGAAGTCACAC | p.Arg319fs |
| 1 | 29254 | 28 | synonymous_variant | *N* | c.981G>A | p.Ser327Ser |
| 1 | 29256 | 28 | frameshift_variant | *N* | c.985delA | p.Thr329fs |
| 15 | 26831 | 170 | frameshift_variant | *M* | c.309_310insAA | p.Ala104fs |
| 13 | 26822 | 146 | synonymous_variant | *M* | c.300C>T | p.Phe100Phe |
| 12 | 26824 | 146 | missense_variant | *M* | c.302G>C | p.Arg101Thr |
| 12 | 26828 | 157 | synonymous_variant | *M* | c.306G>T | p.Leu102Leu |
| 9 | 26837 | 240 | frameshift_variant | *M* | c.316delA | p.Thr106fs |
| 4 | 26701 | 378 | frameshift_variant&missense_variant | *M* | c.179_180delTAinsC | p.Val60fs |
| 4 | 26767 | 1805 | missense_variant | *M* | c.245T>C | p.Ile82Thr |
| 1 | 26692 | 13 | frameshift_variant | *M* | c.171delA | p.Leu57fs |
| 1 | 26705 | 387 | synonymous_variant | *M* | c.183T>C | p.Thr61Thr |
| 1 | 26793 | 23 | frameshift_variant | *M* | c.272_275delTGTG | p.Met91fs |
| 1 | 26931 | 141 | frameshift_variant | *M* | c.410_411delAA | p.Glu137fs |
| 1 | 27165 | 371 | frameshift_variant | *M* | c.644dupA | p.Asp215fs |
| 1 | 27170 | 750 | synonymous_variant | *M* | c.648T>C | p.Asn216Asn |
| 8 | 210 | 24 | intergenic_region | CHR_START-ORF1ab | n.210G>T |  |
| 8 | 241 | 24 | intergenic_region | CHR_START-ORF1ab | n.241C>T |  |
| 2 | 71 | 252 | intergenic_region | CHR_START-ORF1ab | n.71C>A |  |

Table S2: The 28 Structural variations, length, start position and support reads

| **Sample_ID** | **Structural Variations** | **Length (bp)** | **Start Position** | **Support reads** |
| --- | --- | --- | --- | --- |
| sample96 | DEL | -486 | 11339 | 10 |
| sample85 | DEL | -234 | 22114 | 20 |
| sample2 | INS | 171 | 24375 | 30 |
| sample96 | INS | 176 | 24375 | 50 |
| sample40 | INS | 167 | 24422 | 43 |
| sample75 | INS | 174 | 24429 | 23 |
| sample62 | INS | 167 | 24466 | 14 |
| sample30 | INS | 167 | 24466 | 21 |
| sample28 | INS | 174 | 24467 | 10 |
| sample17 | INS | 169 | 24472 | 14 |
| sample05 | INS | 195 | 24472 | 11 |
| sample66 | INS | 165 | 24475 | 13 |
| sample78 | INS | 198 | 24495 | 11 |
| sample54 | INS | 167 | 24505 | 17 |
| sample96 | DEL | -196 | 24510 | 21 |
| sample40 | DEL | -248 | 24542 | 18 |
| sample30 | DEL | -172 | 24593 | 15 |
| sample40 | INS | 142 | 9119 | 34 |
| sample85 | INS | 148 | 9142 | 12 |
| sample62 | INS | 146 | 9143 | 19 |
| sample3 | INS | 143 | 9161 | 17 |
| sample17 | INS | 143 | 9164 | 14 |
| sample2 | INS | 143 | 9164 | 26 |
| sample30 | INS | 143 | 9170 | 20 |
| sample49 | INS | 147 | 9174 | 15 |
| sample75 | INS | 147 | 9185 | 18 |
| sample96 | INS | 147 | 9189 | 25 |
| sample28 | INS | 142 | 9196 | 11 |
| sample14 | INS | 140 | 9203 | 11 |
| sample2 | DEL | -599 | 9244 | 11 |
| sample62 | DEL | -599 | 9244 | 12 |


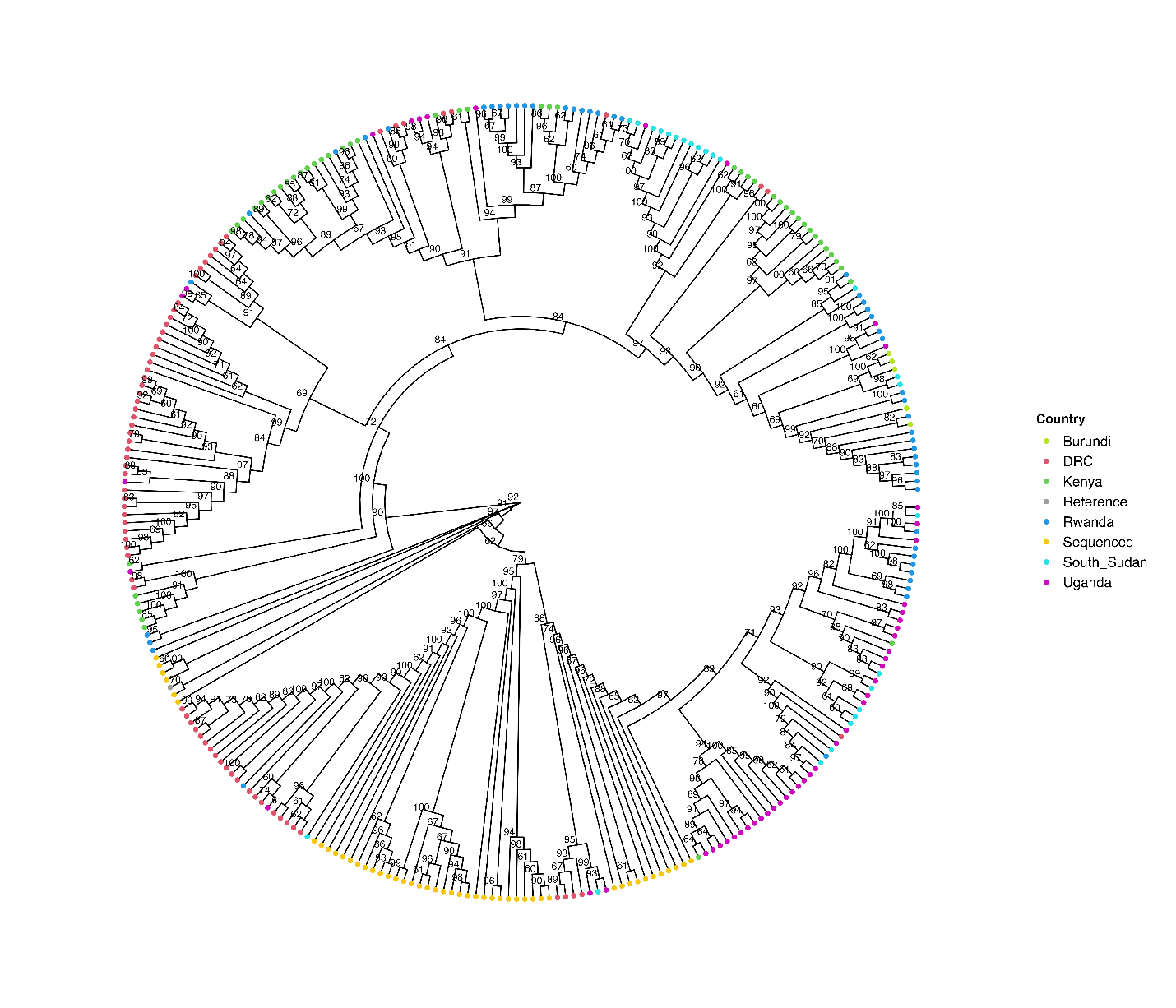


**Fig S1.** Maximum likelihood phylogenetic tree with Bootstrap values depicting genetic relatedness between the sequenced SARS-CoV-2 samples in this study (i.e., ‘Sequenced’) and the rest of East Africa. Nodes are *colored* per country. The 49 samples from Uganda clustered and shared a root with other Ugandan sequences from GISAID.
